# A hand‐targeted auxiliary personal protective equipment for intervention of fomite transmission of viruses

**DOI:** 10.1002/btm2.10411

**Published:** 2022-09-29

**Authors:** Justin Kok Soon Tan, Shang Wei Song, Jialiu Zeng, Chih Hung Lo

**Affiliations:** ^1^ Department of Biomedical Engineering National University of Singapore Singapore Singapore; ^2^ The N.1 Institute for Health National University of Singapore Singapore Singapore; ^3^ Lee Kong Chian School of Medicine Nanyang Technological University Singapore Singapore

**Keywords:** artificial human skin, polyurethane, SARS‐CoV‐2, spike protein, surface contamination, virus‐like particles, wearable device

## Abstract

In COVID‐19, fomite transmission has been shown to be a major route for the spreading of the SARS‐CoV‐2 virus due to its ability to remain on surfaces for extended durations. Although glove wearing can mitigate the risk of viral transmission especially on high touch points, it is not prevalent due to concerns on diversion of frontline medical resources, cross‐contamination, social stigma, as well as discomfort and skin reactions derived from prolonged wearing. In this study, we developed FlexiPalm, a hand‐targeted auxiliary personal protective equipment (PPE) against fomite transmission of viruses. FlexiPalm is a unique palmar‐side hand protector designed to be skin‐conforming and transparent, fabricated from medical‐grade polyurethane transparent film material as a base substrate. It serves primarily as a barrier to microbial contamination like conventional gloves, but with augmented comfort and inconspicuousness to encourage a higher public adoption rate. Compared to conventional glove materials, FlexiPalm demonstrated enhanced mechanical durability and breathability, comparable hydrophobicity, and displayed a minimal adsorption of SARS‐CoV‐2 spike protein and virus‐like particles (VLP). Importantly, FlexiPalm was found to bind significantly less viral protein and VLP than artificial human skin, confirming its ability to reduce viral contamination. A pilot study involving participants completing activities of daily living showed a high level of comfort and task completion, illustrating the usability and functionality of FlexiPalm. Moreover, we have demonstrated that surface modification of FlexiPalm with microtextures enables further reduction in viral adsorption, thereby enhancing its functionality. An effective implementation of FlexiPalm will bolster PPE sustainability and lead to a paradigm shift in the global management of COVID‐19 and other infectious diseases in general.

## 1 INTRODUCTION

COVID‐19 is a pandemic that has affected more than 550 million people worldwide and caused more than 6 million deaths across the world.^1^ Despite diligent measures to control the spread of the SARS‐CoV‐2 virus, the test positivity rate remains high in the community. While the main route of SARS‐CoV‐2 transmission is via close contact and aerosols,^2^ the extent of fomite transmission through inanimate surfaces also plays an important role.^3^ SARS‐CoV‐2 virus particles have been shown to remain viable for 2 to 3 days on smooth surfaces such as stainless steel, plastic, glass, as well as at lower temperature and humidity.^4^, ^5^ The SARS‐CoV‐2 virion has also been shown to remain stable and contagious for up to 9 hours on human skin.^6^ Furthermore, SARS‐CoV‐2 virus particles can easily be detected on high touch points such as shopping carts and baskets.^7^ Hospital wards, both intensive care and general, have also been found to harbor extensive viral contamination, with 56.7% of rooms having at least one environmental surface contaminated with SARS‐CoV‐2 viruses.^8^ High touch surface contamination was found in 66.7% of rooms occupied by patients in the first week of illness, and in 20% for patients beyond the first week of illness.^8^ These studies indicate that there is a high possibility of viral transmission and infection through surface contact.

Recent studies have proposed that personal protective equipment (PPE) appropriate for protection against SARS‐CoV‐2 should not only consist of protective masks but also gloves, to significantly reduce the risk of infection.^9^, ^10^ However, glove usage has not been widely practiced in countries across the world, with only a few countries adopting the use of gloves, such as the United States of America (USA),^11^ Hong Kong,^12^ and Malaysia^13^; even in these countries the adoption rate is modest. For some, glove usage is discouraged due to the diversion of medical resources from the frontline, as well as complacency and contamination due to improper glove removal.^14^ Other reasons that can lead to low adoption rate of gloves include social stigma associated with excessive protection^15^ as well as discomfort and skin reactions derived from prolonged wearing of tight‐fitting gloves.^16^ We also conducted a survey which confirmed that a substantial proportion of participants indeed have these concerns. Hence, this necessitates an alternative form of hand protection, which circumvents these concerns.

**FIGURE 1:**
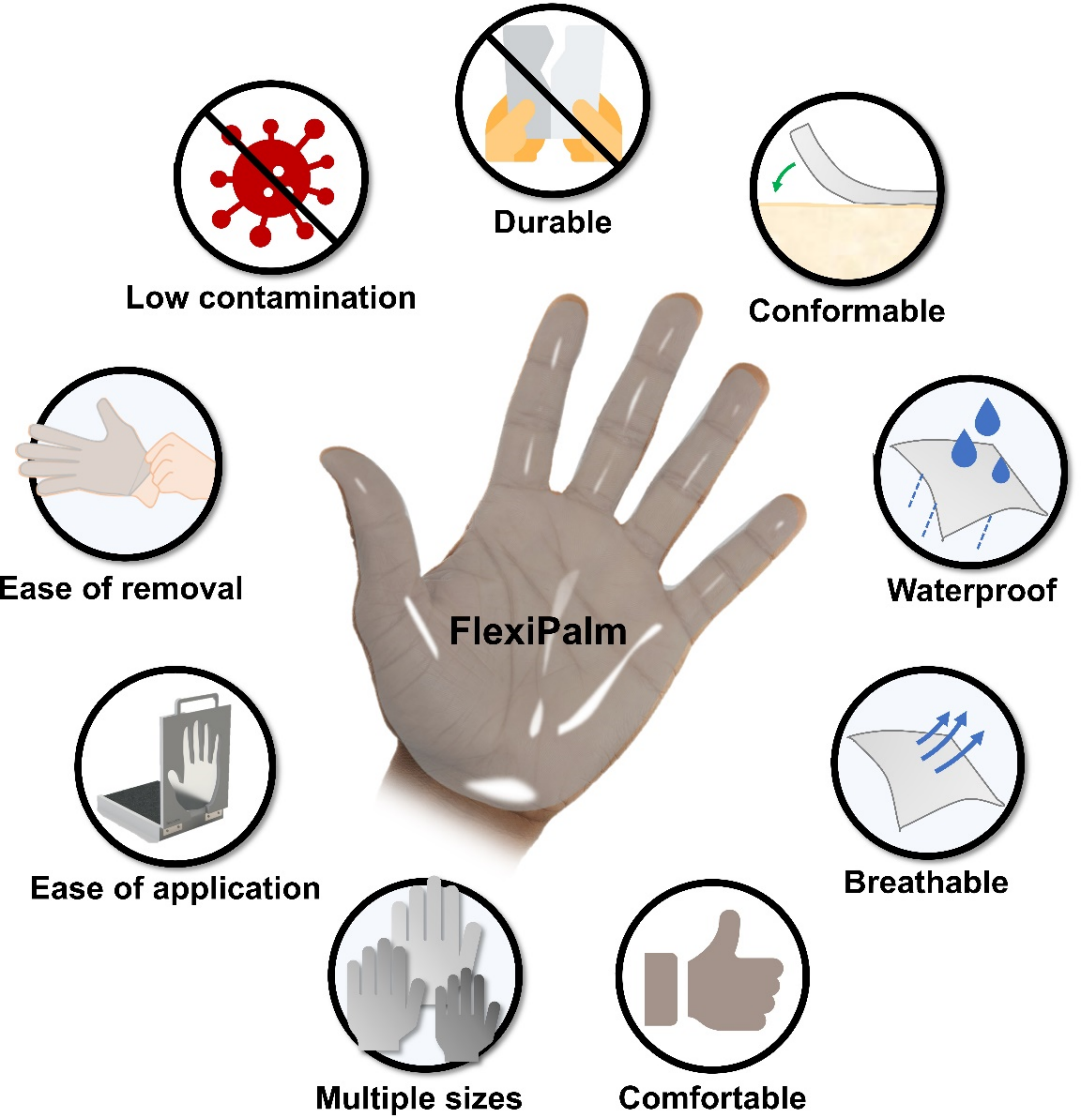
Overview of FlexiPalm properties and design criteria. FlexiPalm (center) is a hand‐targeted auxiliary PPE with a unique palmar‐side hand protection against fomite transmission of viruses. FlexiPalm is designed to satisfy the following criteria: (1) durable, (2) conformable, (3) waterproof, (4) breathable, (5) comfortable, (6) multiple sizes, (7) ease of application, (8) ease of removal, and (9) low contamination (properties surrounding the FlexiPalm). PPE, personal protective equipment

Here, we designed FlexiPalms, the first palm‐directed hand PPE to protect against fomite transmission of viruses. Previous studies have demonstrated the predominance of palmar hand‐to‐face and hand‐to‐mouth contacts as opposed to the dorsal side.^17^, ^18^ Separately, the palmar side of the hand has been observed to make more frequent contact with surfaces than the back of the hand.^19^ Furthermore, other studies have alluded to the lower usage of the dorsal surface of gloves.^20^, ^21^ In view of this, the FlexiPalms are intended to protect the palmar side of the hands, which also reduces material usage and boosts hand PPE sustainability. However, by removing the dorsal side of support, this necessitates an adhesive layer to stick the FlexiPalms to the users' palms directly. Furthermore, there is a need for the following design criteria: (1) good mechanical properties with high elasticity and durability for prolonged usage, (2) hydrophobic surface that is waterproof to reduce aerosol contact with virus particles, (3) high permeability for improved breathability resulting in enhanced comfort, (4) user‐friendly design that allows for ease of wearing and removal, and (5) low viral adsorption with minimal chances of contamination (Figure 1).

To achieve these design criteria, the FlexiPalms were fabricated from a medical‐grade polyurethane (PU) transparent film material as a base substrate.^22^ This material boasts high water vapor permeability, which prevents moisture buildup and bacterial infections on the skin surface.^23^ In addition, the acrylate adhesive minimizes skin damage and can stick to the skin for long durations.^24^, ^25^ We benchmarked the PU material used to fabricate the FlexiPalms against other conventional glove materials, including nitrile, latex, and low‐density polyethylene (LDPE) to ascertain its suitability to be used for hand protection. The PU material demonstrated enhanced mechanical durability, superior breathability, and minimal adsorption of SARS‐CoV‐2 spike protein and virus‐like particles (VLP). Importantly, we confirmed FlexiPalm's ability to reduce viral contamination by showing that it binds significantly less VLP and viral protein than artificial human skin. We also conducted a pilot study to collect user feedback from the execution of tasks while donning the FlexiPalms. Results from our pilot study showed that FlexiPalms display good functionality and attain a high level of receptiveness from the participants as a new form of PPE for hand protection. Furthermore, we conducted a proof‐of‐concept demonstration of the surface modifiability of FlexiPalm with microtextures, which enabled additional reduction in the adsorption of viral protein and VLP, thereby enhancing its functionality.

## 2 MATERIALS AND METHODS

### 2.1 Market survey to gather public perception on hand protection

A survey was conducted to obtain feedback from the general population regarding their view on hand protection during COVID‐19 pandemic. A total of 130 participants from 10 different countries including Singapore, USA, China, United Kingdom, Malaysia, India, Indonesia, Sweden, South Korea, and Israel, participated in the survey. Briefly, we gathered participants' views towards the COVID‐19 situation, including their attitude towards the pandemic, how the pandemic has affected their lives, as well as their risks and potential exposure to the SARS‐CoV‐2 virus. From there, we followed to ask the participants about their frequency of masks and gloves usage, as well as other hygiene routines such as hand washing and usage of sanitizers and disinfectants. We then specifically asked about the participants' perception of glove wearing as a form of protection during the pandemic, including their awareness of the SARS‐CoV‐2 virus remaining on surfaces for prolonged durations and possible fomite transmission, attitude towards using gloves, concerns with glove usage, as well as receptiveness to adopting a new form of hand PPE (FlexiPalm). This study was conducted following the study protocol approved by the Institutional Review Board of National University of Singapore (NUS‐IRB‐2020‐816).

### 2.2 Polymeric materials for benchmarking studies

Six samples were characterized in this study: (i) Tegaderm transparent film roll (3M, MN, USA), (ii) Opsite Flexifix (Smith & Nephew Co, London, UK), (iii) Chengxingsis transparent film dressing (Chengxingsis, China), (iv) KleenGuard G20 nitrile glove (Kimberly‐Clark, WI, USA), (v) Biomedia latex glove (Biomedia, Singapore), and (vi) LDPE transparent film (The Glad Products Company, USA). The six materials are hereafter referred to as “TGM PU,” “OPS PU,” “CXG PU,” “Nitrile,” “Latex,” and “LDPE,” respectively. The surface modification of the FlexiPalm was performed by casting TGM PU onto aluminum oxide sandpapers of varying grit sizes (800, 360, and 60 grit) to create the microtextured surface.

### 2.3 Fabrication of FlexiPalms

**FIGURE 2:**
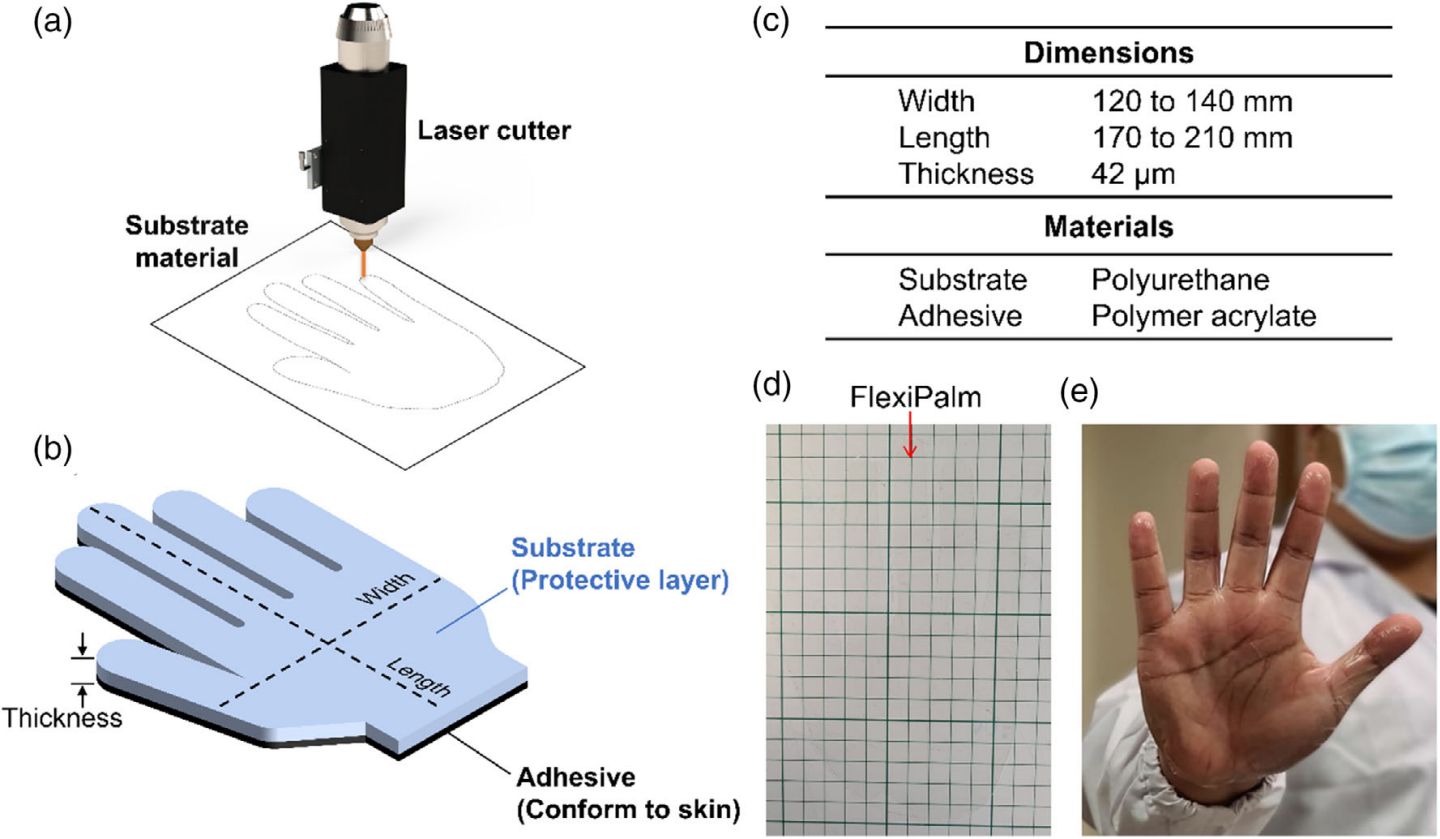
Design and fabrication of FlexiPalms. (a) FlexiPalms are fabricated from the substrate material by laser cutting. (b) Schematic of the substrate and adhesive layers of the FlexiPalm with the corresponding dimensions. (c) Dimensions and materials (substrate and adhesive) of the fabricated FlexiPalms. (d) The FlexiPalm is demonstrated to be transparent and inconspicuous. (e) The application of FlexiPalm onto the hand of a human subject

FlexiPalms were illustrated using AutoCAD (Autodesk, CA, USA) and cut from TGM PU with a 9.3 μm pulsed laser cutter (VLS2.3, Universal Laser Systems, Inc., USA) (Figure 2a). The engraving power and speed were set at 30% and 70 mm/s, while the laser focusing depth (*z*‐axis location) was set to 13.6 mm. The TGM PU comprises a PU backing and a medical‐grade acrylate adhesive (Figure 2b,c). Three sizes were fabricated—small, medium, and large—with palm widths of 120, 130, and 140 mm and lengths of 170, 190, and 210 mm, respectively (Figure 2c). Furthermore, the palm shape was cut with perforation lines for ease of separation from the backing material. FlexiPalms were demonstrated to be transparent (Figure 2d) and inconspicuous when worn on users' hands (Figure 2e).

### 2.4 Fabrication and usage of applicator

For ease of donning of the FlexiPalms, we further designed an applicator prototype to allow fast alignment and contouring of FlexiPalms to the users' hands (Figure S1). The lid with a palm‐shaped cut‐out as well as the base to contain a memory foam block were 3D‐printed by fused deposition modeling (UP BOX, Tiertime, China) using acrylonitrile butadiene styrene as the primary material (Figure S1A). To don the FlexiPalms, the perforated peelable backing material was removed and pasted onto the lid with careful alignment with the palm‐shaped cut‐out (Figure S1B), with the adhesive layer exposed for application (Figure S1C). The FlexiPalm was pressed through the palm‐shaped opening along the perforated line by the user (Figure S1D). Pressure was exerted onto the FlexiPalm against the memory foam block, which conformed to the user's palm contours. The user's hand was withdrawn from the applicator upon the donning of the FlexiPalm (Figure S1E).

### 2.5 Mechanical characterization

Uniaxial tensile tests were conducted to determine the mechanical properties of the materials. The testing protocol was adapted from ASTM D638. The materials were cut into a 6 mm by 115 mm Dog‐Bone shape and mounted onto the grippers of a tensile tester (Instron 3345, Instron, MA, USA). The extension rate was set at 300 mm/min and the initial load and displacement were zeroed prior to each measurement. The mechanical parameters of tensile strength, Young's modulus, and elongation at break were derived from the generated stress‐strain curves.

### 2.6 Adhesive strength test

Adhesive strength tests were conducted to measure the amount of force required to peel the three different PU transparent film samples from porcine skin. Porcine skin was purchased from a local abattoir and prepared with reference to the preparation guidelines in ASTM F2256. These were subsequently mounted onto an acrylic block, which was secured by the bottom gripper of the Instron tensile tester. The test materials were cut into 24 mm by 300 mm rectangular strips, with the adhesive surfaces at either end anchored to the top gripper and adhered to the porcine skin respectively at an angle of 180°. The extension rate for the machine was set at 300 mm/min and the initial load and displacement were zeroed prior to measurements. The adhesive strength of the characterized materials was derived from the generated load‐extension curve.

### 2.7 Wettability measurements

The wettability of the samples was characterized by the water contact angle (WCA). WCA was measured using the sessile drop method with a 10 μL drop of deionized (DI) water. All WCA data was averaged over measurements at three different locations on the samples. The samples were mounted on the stage of a micro‐manipulator (M3301R, Prime Bioscience, Singapore) and images were captured with a high‐definition camera (XCAM1080PHA, ToupTek, China), and subsequently analyzed using an image analysis software (ImageJ, National Institutes of Health, USA).

### 2.8 Moisture vapor transmission test

Moisture vapor transmission tests were conducted to determine the rate of water vapor flux through a controlled surface area of a material within a specified duration under controlled conditions.^26^ Centrifuge tubes (50 mL size, Fisher Scientific, NH, USA) containing 20 mL of DI water were sealed off with each material stretched over the mouth of the tubes. The initial weights of the tubes were measured with an analytical balance (Mettler Toledo, OH, USA). The tubes were then placed in a convection oven (Thermo Scientific, MA, USA) for 24 hours at a constant temperature of 40°C, after which they were removed from the oven and weighed. The moisture vapor transmission rates (MVTR) were then calculated based on the equation $\left(W_{24}-W_{0}\right)/\left(T\times A\right)$, where T is the duration in the oven, A is area of the mouth of the centrifuge tubes, and $W_{0}$ and $W_{24}$ refer to the weight of the tubes before and after the 24‐hour period.

### 2.9 SARS‐CoV‐2 spike protein and VLP adsorption assays and ELISA assays

SARS‐CoV‐2 recombinant spike protein (Abnova, Taiwan) was reconstituted in phosphate buffered saline (PBS) to a concentration of 10 μg/mL. SARS‐CoV‐2 VLP (Abnova, Taiwan) was resuspended in PBS to a concentration of approximately 10^8^ particles/mL corresponding to approximately 6.8 log unit of 50% tissue culture infectious dose (TCID_50_) per mL. The material samples (10 mm circles) were mounted on the base of a 96‐well microplate (Corning, NY, USA). Two hundred microliters of the spike protein or VLP solution was added to each well and incubated at room temperature for 2 hours. For immunostaining, the incubated samples were washed three times with PBS and incubated with the human spike protein primary antibody (Novus, Singlab, Singapore) overnight at 4°C. Subsequently, the samples were washed with PBS, and incubated with goat antihuman immunoglobulin G (IgG) fluorescein isothiocyanate (FITC)‐tagged secondary antibody (Abcam, UK) at room temperature for 1 hour. The samples were then washed, and the amount of absorbed spike protein was quantified based on fluorescence intensity measured by epi‐illumination (excitation/emission: 490/520 nm) on an inverted microscope (Olympus IX71, Olympus, Japan) at 4× magnification. For the enzyme‐linked immunosorbent assay (ELISA) assays, the samples were preincubated with the SARS‐CoV‐2 recombinant spike protein and VLP and subsequently washed with PBST for 15 minutes on a shaker at 80 RPM. The eluent was assayed for the SARS‐CoV‐2 spike protein and VLP by sandwich ELISA following the manufacturer's protocol (Abnova, Taiwan).

### 2.10 Pilot study to assess the functionality and performance of FlexiPalms

To assess the durability and comfort of the FlexiPalms, a pilot study was conducted on 41 individuals. The inclusion criteria were healthy individuals between the ages of 21 to 65 years. Individuals who are unable to move their upper limb, with preexisting dermatological conditions or with injuries that would render them unable to carry out the tasks in the pilot study were excluded from the study. Written informed consent was obtained from all participants prior to the start of the study. The amount of damage sustained by the FlexiPalms was assessed based on the number of peel‐off areas and classified as “major” (>2 peel‐off areas), “minor” (≤2 peel‐off areas), or “no peel‐off.” The comfort level was determined by users' discretion based on how they felt while performing the tasks. This study was conducted following the study protocol approved by the Institutional Review Board of the National University of Singapore (NUS‐IRB‐2021‐22). Participants of the pilot study were instructed to perform a total of 11 activities of daily living adapted from the Graded Repetitive Arm Supplementary Program (GRASP) rehabilitation program for stroke patients (Task 1A to 7B, Figure S2).^27^ To ascertain if mechanical loading associated with the pilot study activities resulted in attenuated mechanical integrity, used FlexiPalms were removed from participants' hands, from which 50 × 10 mm rectangular strips were cut for post‐loading mechanical characterization.

### 2.11 Skin irritation assay

We performed a skin irritation assay according to the OECD 439 in vitro skin irritation test to check the cytotoxicity on human epidermal tissue induced by the FlexiPalm material for up to 72 hours. Reconstructed human epidermal tissue samples (MatTek, MA, USA) were preincubated with 0.9 mL of assay media in a 6‐well plate for 1 hour, followed by a media change and incubation at 37°C in a 5% CO_2_ incubator for 24 hours. Sample extraction was performed according to ISO 10993‐12 (Preparation of Medical Device Extracts for ISO/TC 194 WG 8 Irritation and Skin Sensitization). Briefly, 6 cm^2^ pieces of TGM PU were suspended in a borosilicate glass tube at 37°C for 72 hours with continuous shaking. For the skin irritation assay, 100 μL of the undiluted medical device extract, negative control (PBS), positive control (0.5% sodium dodecyl sulfate (SDS)), and saline were dosed onto the tissue samples for 18 hours at 37°C in a 5% CO_2_ incubator. Subsequently, the tissue samples were rinsed with sterile PBS thoroughly and blotted dry. Treated tissue samples were transferred into 24‐well plate prefilled with 0.3 mL/well of MTT (3‐[4,5‐dimethylthiazole‐2‐yl]‐2,5‐diphenyltetrazolium bromide) solution and incubated at 37°C in a 5% CO_2_ incubator for 3 hours. The MTT solutions were then aspirated and formazan extraction was carried out by adding 1 mL of isopropanol to each well and shaking for 2 hours. Two hundred microliters of the extracted solutions were then transferred into a 96‐well plate, with 200 μL of isopropanol as blank. Optical density was measured at 570 nm.

### 2.12 Characterization of surface modified TGM PU

The surface morphologies of the microtextured TGM PU samples were imaged by high‐resolution field emission scanning electron microscopy (FE‐SEM) (Regulus 8100, Hitachi, Japan) after gold‐sputtering (20 nm thickness). Surface roughness (standard deviation of the surface topology) was measured by laser scanning microscopy (VK‐X3000, Keyence Corporation, Japan) at a vertical resolution of 1 μm.

### 2.13 Measurement of static coefficient of friction (CoF)

The static CoF of the smooth and microtextured TGM PU samples was measured by the inclined plane testing method ASTM‐G219. 40 × 40 mm sample pairs were cut and mounted onto the platform of a micromanipulator and the base of a 160 g stainless steel sled, respectively. The platform was tilted gradually until the sled first moved. The static CoF was calculated based on the tangent of the tilt angle.

### 2.14 Statistical analysis

Data visualization and statistical analysis were analyzed using Graphpad Prism 9. Results are presented as means ± standard deviations. Unpaired Student's *t*‐test (for parametric samples) and Mann‐Whitney test (for nonparametric samples) were used to analyze the data benchmarking the FlexiPalms to other conventional glove materials. Differences were considered statistically significant for *P* < .05. *P < .05, **P < .01, ***P < .001, ****P < .0001, and ns indicates not significant.

## 3 RESULTS

### 3.1 Public has concerns over glove usage and supports FlexiPalms as a new form of PPE

**FIGURE 3:**
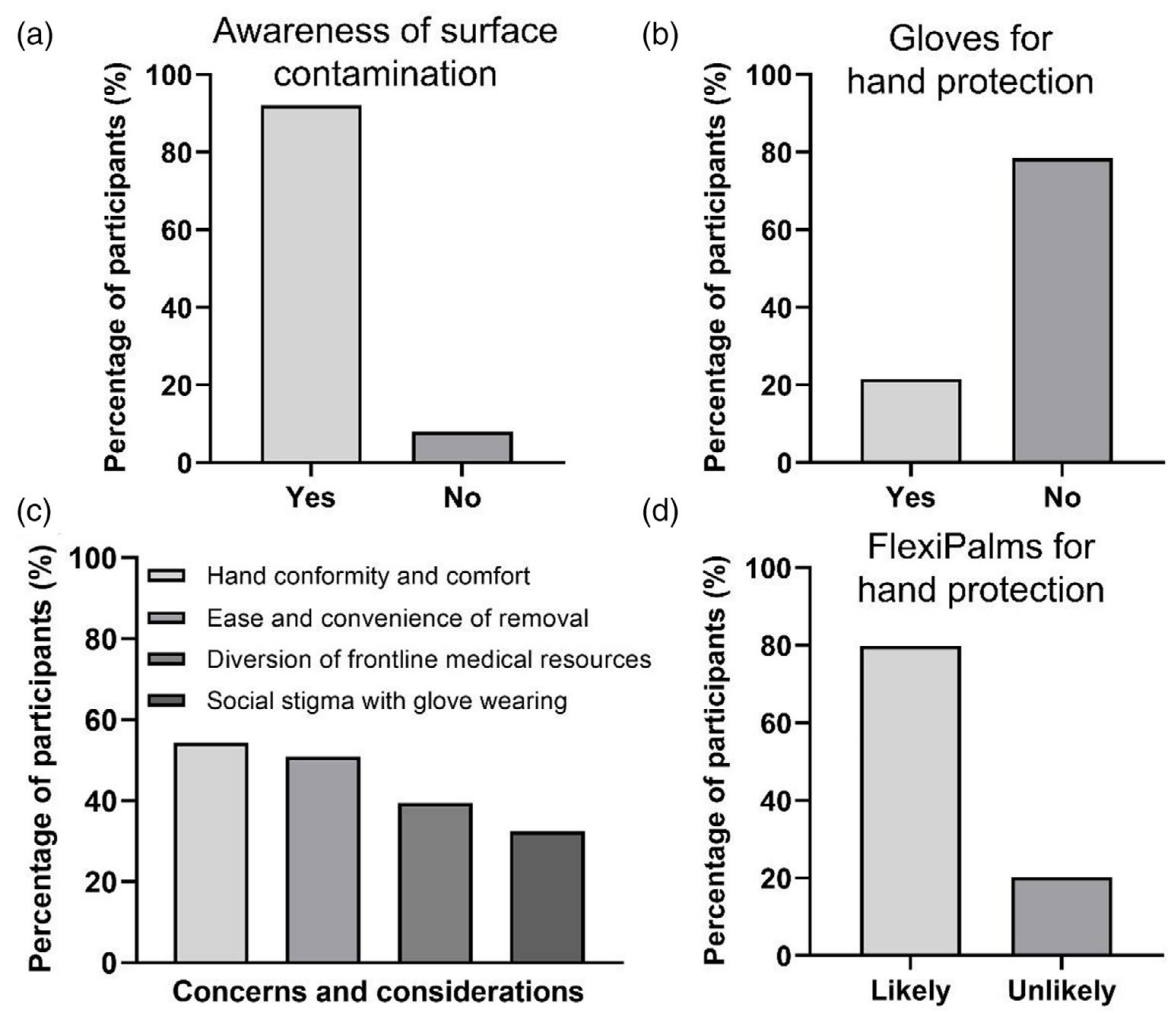
Market survey to understand public concerns over glove usage and their receptiveness to FlexiPalms as a new form of hand PPE. (a) Awareness of surface contamination of virus particles as a way of virus transmission. (b) Public adoption of gloves as a PPE for hand protection. (c) Public concerns and considerations pertaining to glove usage for hand protection. (d) Public receptiveness to FlexiPalms in terms of their likelihood of adopting FlexiPalms as a PPE for hand protection. PPE, personal protective equipment

To gather feedback from the population and to identify the issues associated with glove wearing, we designed a survey questionnaire and distributed it to people worldwide to gather their inputs. We acquired detailed information on sociodemographic characteristics, awareness of SARS‐CoV‐2 transmission via surface contact, glove usage to prevent fomite transmission, concerns with glove usage, factors promoting the wearing of hand PPE, as well as the receptiveness of FlexiPalm as a new form of PPE. We collected survey responses from a total of 130 people from 10 countries. We found that although 92% of the respondents are aware that viruses can spread through surface contacts (Figure 3a), only 22% of them wear gloves as a hand PPE to protect themselves against contracting viruses (Figure 3b). This lack of gloves wearing to prevent fomite transmission suggests that people have concerns with using the currently available hand PPE. Hence, it is important to understand the concerns that people have and the factors that may encourage people to adopt the use of hand PPE to protect themselves. Concerns highlighted in the previous studies^15^, ^16^ were presented to respondents to collect quantitative information on the relative importance of each concern or consideration to understand the general sentiment underscoring the lack of glove usage. We identified that the respondents' main concerns and considerations with using conventional gloves include hand conformability and comfort (54%), ease and convenience of removal (51%), diversion of frontline medical resources (39%), and social stigma associated with glove wearing (32%) (Figure 3c). These highlight the limitations of existing gloves, which necessitates a new form of hand PPE with a novel design to encompass these features. We then presented the respondents with our novel FlexiPalm concept, which was designed to address the aforementioned concerns. Remarkably, 80% of the participants responded that they will likely adopt the use of FlexiPalms (Figure 3d), a striking nearly 60% increase as compared to the current glove usage. Hence, this illustrated the public receptiveness to FlexiPalm and indicated the potential increase in adoption of hand PPE in the community to protect against fomites.

### 3.2 FlexiPalms are fabricated from TGM PU material which exhibits enhanced mechanical properties than conventional glove materials

**FIGURE 4:**
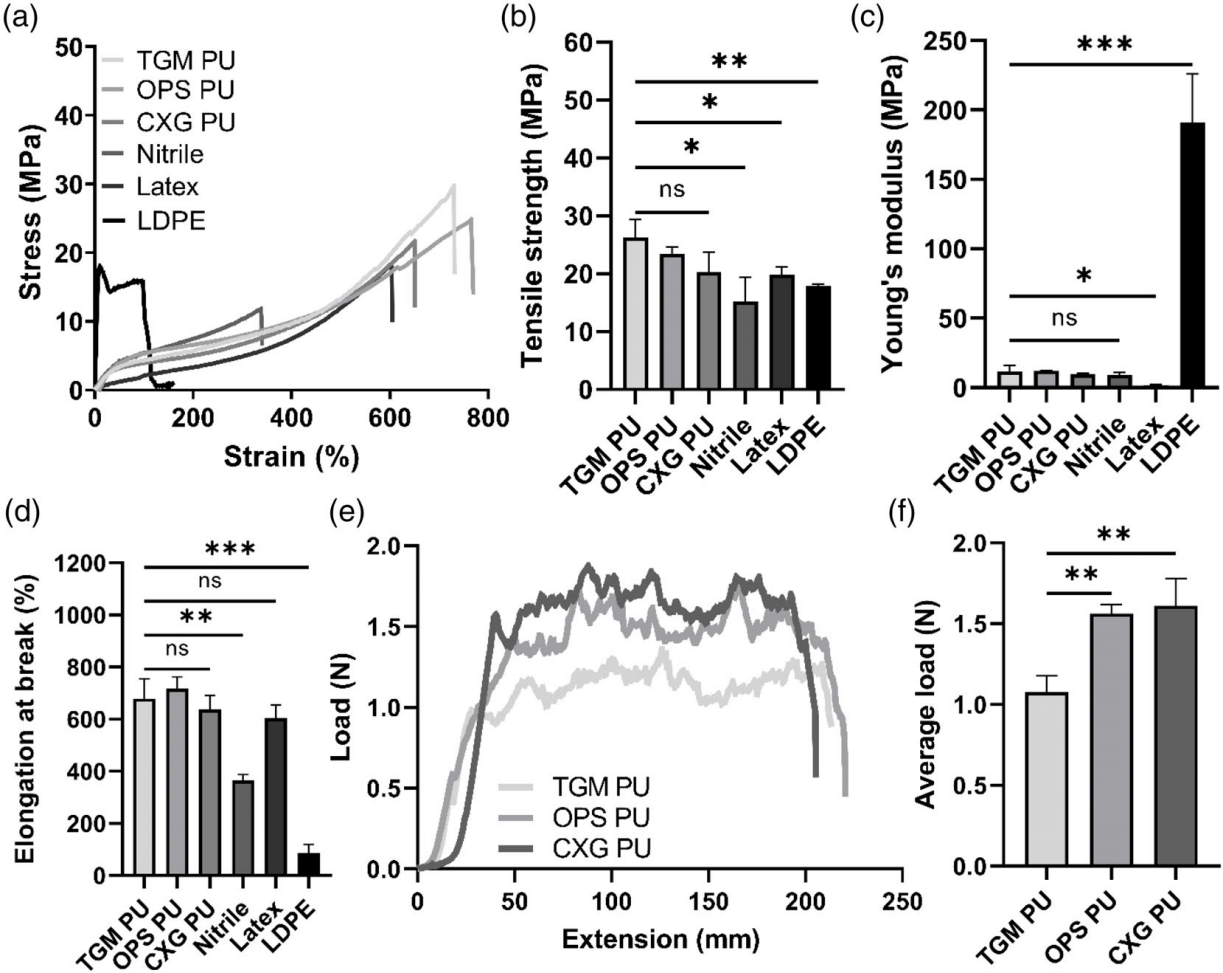
Benchmarking of mechanical properties of PU materials against conventional glove materials for FlexiPalms fabrication. (a) Stress‐strain curves, (b) mechanical strength, (c) Young's modulus, and (d) elongation at break of the characterized materials, including three types of PU transparent films, nitrile, latex, and LDPE. (e) Load‐extension curves and (f) average load during the peel off phase between extension of 50 to 150 mm in (e) of the three PU transparent films. *P < .05, **P < .01, ***P < .001, and ns indicates not significant. LDPE, low‐density polyethylene; PU, polyurethane

The primary function of the FlexiPalm is to serve as a physical barrier against pathogens, similar to gloves. With the design criteria (Figure 1) in mind, we sought to evaluate the three commercially available PU transparent film materials (TGM PU, OPS PU, and CXG PU) to see if their mechanical properties emulated those of conventional glove materials (nitrile, latex, and LDPE). Uniaxial tensile testing was conducted to measure their durability based on the ultimate tensile strength, stiffness based on the Young's modulus, and conformability based on the extensibility. From the stress‐strain curves, LDPE displayed a standard linear elastic deformation behavior, while nitrile, latex, and all three PU materials exhibited the typical strain‐hardening response associated with polymeric elastomers after their yielding point (Figure 4a).^28^

First, there were no significant differences observed between the three PU materials in terms of their tensile strength, Young's modulus, and elongation (Figure 4b‐d). The PU materials exhibited the highest tensile strengths (TGM PU: 26.22 ± 3.09 MPa, OPS PU: 23.38 ± 1.23 MPa, CXG PU: 20.26 ± 3.39 MPa) that were higher than nitrile (15.16 ± 4.18 MPa), latex (19.78 ± 1.32 MPa), and LDPE (17.90 ± 0.29 MPa), which alludes to their superior strength and durability (Figure 4b). The Young's moduli of the PU materials (TGM PU: 11.47 ± 4.55 MPa, OPS PU: 11.99 ± 0.19 MPa, CXG PU: 9.57 ± 0.75 MPa) were comparable to nitrile (9.36 ± 1.60 MPa), higher than latex (1.70 ± 0.51 MPa), and markedly lower than LDPE (190.66 ± 35.49 MPa) (Figure 4c). This warrants further testing to ascertain if PU materials restrict hand mobility and reduce wearer dexterity. Furthermore, the PU materials were measured to have longer elongations at break (TGM PU: 678.07 ± 77.40%, OPS PU: 716.87 ± 46.01%, CXG PU: 636.89 ± 54.06%) than nitrile (363.35 ± 23.42%), latex (603.98 ± 50.19%) and LDPE (85.46 ± 33.33%) (Figure 4d), which demonstrates its conformability to the contours of the palm. Overall, these results highlighted that the PU materials exhibit enhanced mechanical properties to conventional glove materials.

To confirm that FlexiPalm can be applied directly onto human skin and safely peeled off, we measured the peel strength of the adhesive layers of the three PU materials from the horizontal region of the load‐extension curve obtained from the 180° peel test (Figure 4e). The load required to remove TGM PU (1.10 ± 0.07 N) from the porcine skin is significantly lower than OPS PU (1.56 ± 0.06 N) and CXG PU (1.61 ± 0.17 N) (Figure 4f). With TGM PU's good skin compatibility from the adhesive strength test, together with its superior mechanical properties (high durability and conformability as well as comparable stiffness), we have demonstrated its suitability as a substrate for FlexiPalms. TGM PU was hence used for subsequent FlexiPalms fabrication, characterizations, and testing in the pilot study.

### 3.3 TGM PU‐based FlexiPalms are water repellent and provide superior breathability

**FIGURE 5:**
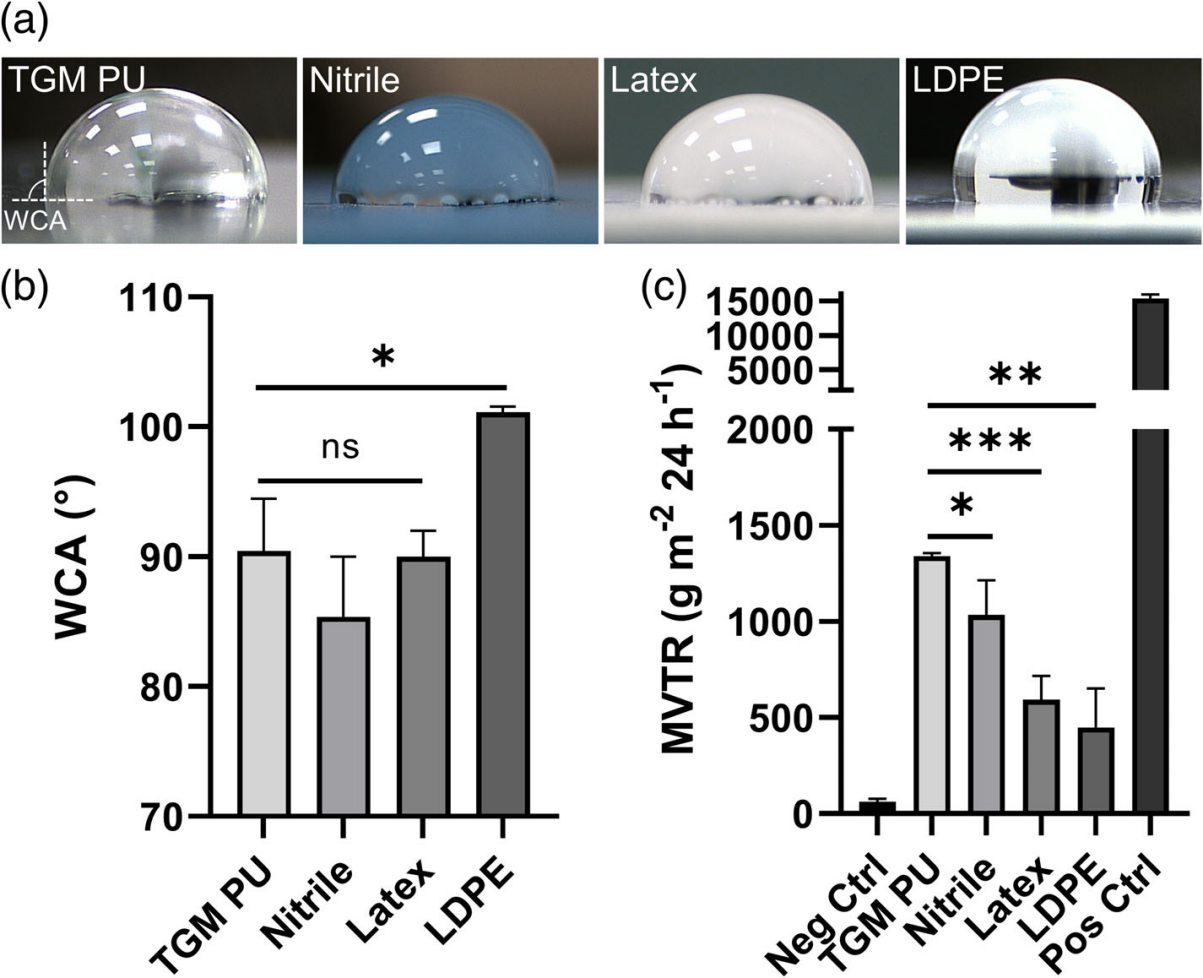
Comparison of the wettability and breathability of TGM PU and conventional glove materials. (a) Images of the WCA measurements of TGM PU, nitrile, latex, and LDPE using the sessile drop method. Dashed lines indicate the material surface and the three‐phase (solid‐air‐liquid) contact line within which the WCA is subtended. (b) Quantified results of the WCA measurements for the four tested materials. (c) Moisture vapor transmission test to measure the breathability of FlexiPalm based on the amount of water vapor that can penetrate the PU transparent film over a fixed duration. *P < .05, **P < .01, ***P < .001, and ns indicates not significant. LDPE, low‐density polyethylene; PU, polyurethane; WCA, water contact angle

We measured the wettability of the TGM PU against the conventional glove materials using the sessile drop technique. TGM PU has a WCA of 90.46 ± 4.00° that exceeds 90°, indicating its hydrophobicity which is comparable to nitrile (85.37 ± 4.64°) and latex (89.08 ± 0.49°), but lower than LDPE (101.11 ± 0.43°) (Figure 5a,b). Apart from water repellency, we assessed the TGM PU material for its breathability to ensure the comfort of the users' hands. Our results showed that TGM PU has a much higher moisture vapor penetrability (MVTR of 1340.84 ± 15.59 g m^−2^ 24 h^−1^) as compared to nitrile (MVTR of 1034.88 ± 209.70 g m^−2^ 24 h^−1^), latex (MVTR of 593.93 ± 123.72 g m^−2^ 24 h^−1^), and LDPE (MVTR of 449.95 ± 202.63 g m^−2^ 24 h^−1^) (Figure 5c), illustrating its superior breathability and suitability as a hand PPE as compared to other conventional glove materials.

### 3.4 TGM PU‐based FlexiPalms offer improvement over medical gloves in minimizing SARS‐CoV‐2 spike protein and VLP adsorption

**FIGURE 6:**
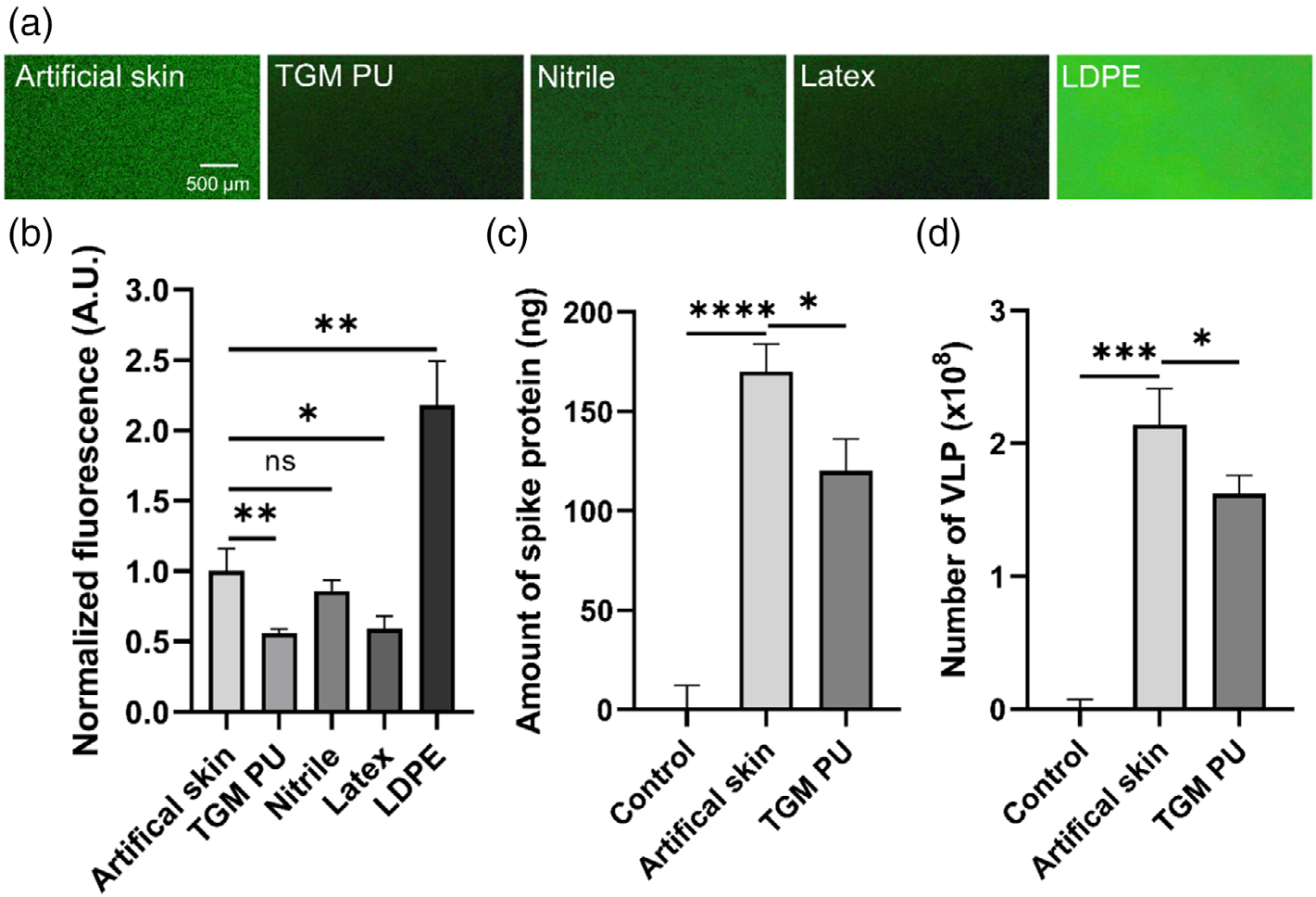
Adsorption assays to quantify the binding propensity of SARS‐CoV‐2 spike protein and VLP onto artificial human skin and tested materials. (a) Epi‐illumination images of SARS‐CoV‐2 spike protein‐FITC adsorbed on the respective materials over a 2‐hour incubation period. (b) Quantification of the amount of bound SARS‐CoV‐2 spike protein in (a) based on FITC fluorescence intensity (A.U. indicates arbitrary unit). (c and d) The amount of SARS‐CoV‐2 spike protein (c) and VLP (d) recovered in the eluent after being rinsed off from the artificial skin and the TGM PU material (FlexiPalms) as quantified by ELISA. *P < .05, **P < .01, ***P < .001, ****P < .0001, and ns indicates not significant. PU, polyurethane; VLP, virus‐like particles

In order to determine the adsorption propensity of viral protein onto the different hand PPE, we incubated TGM PU and conventional glove materials with SARS‐CoV‐2 spike protein solutions and measured their adsorption and binding capability using fluorescent‐tagged antibodies. To fulfill its criteria of reducing viral contamination, we compared the protein binding against an artificial human skin which effectively mimics the surface properties of human skin.^29^ First, TGM PU (0.56 ± 0.03 arbitrary units, A.U.) binds significantly less spike protein than artificial human skin (1.00 ± 0.16 A.U.) based on their normalized fluorescence intensity (Figure 6a,b). There was a comparable amount of spike protein adsorption between TGM PU, nitrile (0.86 ± 0.08 A.U.), and latex (0.59 ± 0.09 A.U.), but much lower than LDPE (2.18 ± 0.31 A.U.) (Figure 6a,b) with the TGM PU having the lowest adsorption value. This was confirmed through ELISA which similarly showed a 30% reduction in the spike protein bound to the TGM PU (120 ng) as compared to the artificial skin (170 ng) (Figure 6c). To mimic the interaction between SARS‐CoV‐2 virus and FlexiPalm or with human skins, we further investigated the binding of SARS‐CoV‐2 VLP, which contain other viral components including membrane, nucleocapsid, spike, and envelope proteins, to TGM PU and the artificial skin. Similarly, TGM PU (1.62 ± 0.14 × 10^8^ particles) bound 25% less VLP compared to the artificial skin (2.14 ± 0.27 × 10^8^ particles) (Figure 6d). This validates the protective function of FlexiPalms through the attenuation of viral adsorption.

### 3.5 Participants favored FlexiPalms over conventional gloves in pilot study

**FIGURE 7:**
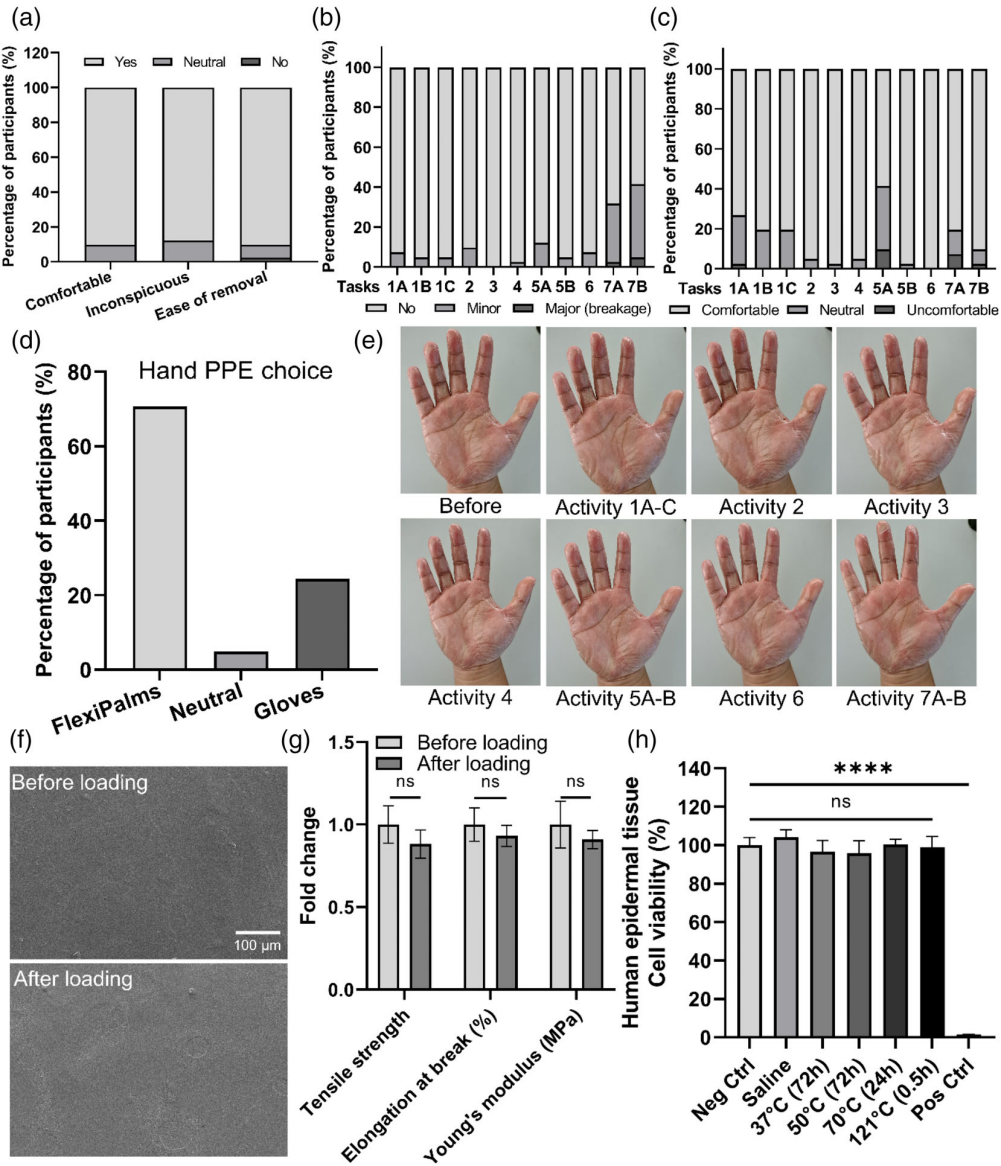
Pilot study to assess the functionality and performance of FlexiPalm. (a) Participants' assessment of comfort, inconspicuousness, and ease of removal of FlexiPalms. (b) Scoring of the conditions of FlexiPalms upon the completion of the tasks assigned to the participants. (c) Scoring of the comfort of FlexiPalms throughout the completion of the tasks assigned to the participants. (d) Participants' preference of hand PPE for protection against fomite transmission of viruses. (e) Images of a participant donning on FlexiPalm before and after performing the activities in the pilot study. (f) FE‐SEM images of the surface of FlexiPalm before and after loading. (g) Mechanical properties of FlexiPalm before and after loading. (h) Characterization of the skin irritability of FlexiPalm by measuring the cell viability of the reconstructed human epidermal tissue. ****P < .0001 and ns indicates not significant. FE‐SEM, field emission scanning electron microscopy

Having completed the materials and biological testing for the FlexiPalms, we went on to find out more about the usability and functionality of our FlexiPalm prototype by conducting a pilot study on 41 participants. We asked them to perform a series of activities of daily living adapted from the GRASP rehabilitation program for stroke patients (Task 1A to 7B, Figure S2A). We first asked the participants about the comfort level and conspicuousness of the FlexiPalms. All participants rated these two aspects as “comfortable/inconspicuous” and “neutral,” with 90.2% and 87.8% of the participants commenting that the initial wearing of FlexiPalms was comfortable and inconspicuous to the eye respectively, and none of them rated “uncomfortable/conspicuous” (Figure 7a). To determine the ease of removal of the FlexiPalms, we asked participants to rate how they felt as they peeled off the FlexiPalms. The majority of the participants (90.2%) felt that the process of FlexiPalms removal was “easy,” whereas 7.3% felt “neutral” and 2.5% felt “difficult” (Figure 7a). High ease of removal, together with the demonstrated water repellency and minimized viral adsorption, will alleviate cross‐contamination with the usage of FlexiPalms.^30^

We also assessed the participants' perception of FlexiPalms by evaluating both their degree of completion of task activities and their comfort level while performing each task. Among the 11 different tasks (Figure S2B), almost all the users were able to complete the tasks with no peel‐off or some minor peel‐off (Figure 7b). Of note, no rupture and breakage of FlexiPalms were observed during the user study. Users faced the most challenge in completing tasks 7A and 7B, which correspond to wringing a dry towel or wet towel, respectively, where about 30% to 35% of the participants completed the task with minor peel‐off, with 2% to 5% of major peel‐off (Figure 7b). From the user feedback, we established that this was mainly due to the loose unadhered edges of the FlexiPalms entangling with the towel, resulting in peel‐off during the wringing motion. Interestingly, when the FlexiPalms were adhered snugly to the hand, no peel‐off was observed upon the completion of these tasks. This suggests that the peel‐off was associated with improper donning of FlexiPalms which results in imperfect adherence. This limitation may be averted with refinement to the application procedure.

For the comfort level, more than 90% of the participants felt that task completion was “comfortable” and “neutral,” indicating no impediment with hand motion and dexterity. The majority of the participants further commented that the FlexiPalms felt comfortable and breathable. However, in Tasks 1 and 5A which require a high level of dexterity and maneuverability (eg, writing and fiddling with small items), 20% to 30% and 2% to 10% of participants rated “neutral” and “uncomfortable” respectively, compared to other tasks (Figure 7c). In addition, 7% to 12% of the participants rated Tasks 7A and 7B as “neutral” (Figure 7c), which may be attributed to their elevated incidence of peel‐off.

Notably, in the context of wearing the FlexiPalms for daily activities for protection against fomite transmission of viruses, 70.7% of participants preferred the FlexiPalms due to better comfort, higher tactile sensitivity, increased breathability, and conforming like a second skin. Some participants (24.4%) preferred medical gloves due to their smooth surface as they found that there was increased friction between FlexiPalms worn on both palms, while 4.9% remained neutral (Figure 7d). One potential reason for this is the adhesion of PU with other PU surfaces,^31^, ^32^ which can be attributed to the hydrogen bonds formed by polar functional groups such as isocyanate (R—N=C=O) and polyol (R—OH) on the TGM PU surface with other polymeric materials containing similar functional groups at very short distances.^33^ Overall, the results from the pilot study show that FlexiPalms satisfied the criteria to be considered as an alternative hand‐targeted PPE to conventional gloves.

To verify the absence of mechanical integrity impairment post wearing, we measured the mechanical properties of the FlexiPalms after being subjected to mechanical loading, including bending, compressive, and shear stresses applied by the participants' hands and the test objects during the execution of each of the activities. First, from visual inspection there was no wear and tear on the FlexiPalms at the end of the pilot study tasks, except for some creasing observed after activity 7 which was not due to rupture but to peel‐off near the edges (Figure 7e). In addition, FE‐SEM images of the worn FlexiPalms did not reveal any noticeable differences in the surface morphology (Figure 7f). Importantly, our quantitative assessment showed no significant differences in the tensile strength, elongation, and Young's modulus between the loaded and unloaded FlexiPalms (Figure 7g and Figure S3A‐C).

After doffing the FlexiPalms, users reported a general ease of removal with no skin irritation. However, the timeframe for the pilot study was short and might not have been capable of capturing the possible adverse skin reactions stemming from long‐term usage. Therefore, we conducted a skin irritation test with reconstructed human epidermal tissue samples which showed the absence of skin damage (>95% cell viability of the human epidermal tissue) when in contact with the FlexiPalms (Figure 7h), consistent with the users' feedback in the pilot study and confirming their suitability for long‐term usage.

### 3.6 Surface modification of FlexiPalms for enhanced functionality

**FIGURE 8:**
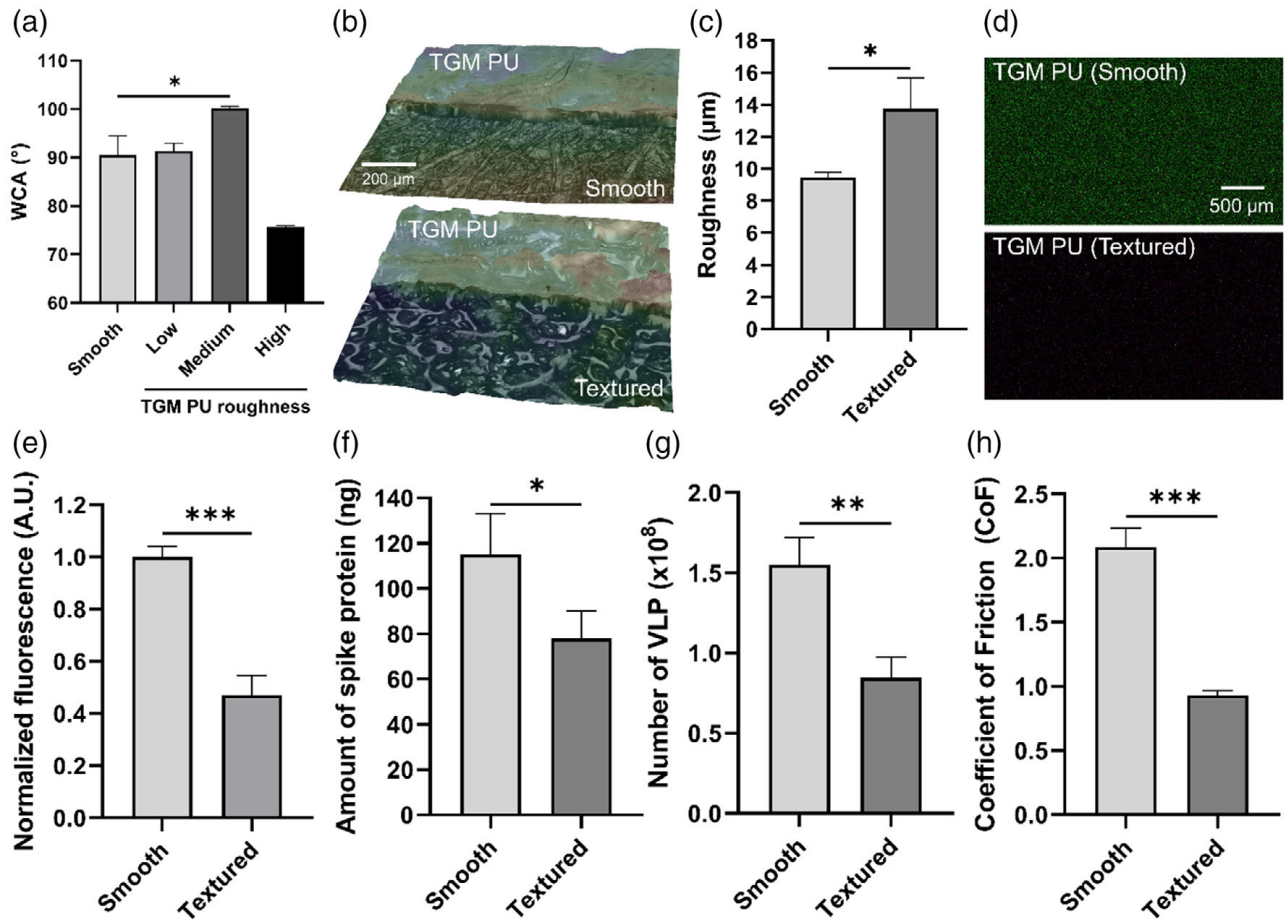
Surface modification of TGM PU for enhanced functionality of FlexiPalm. (a) WCA measurements of TGM PU and surface‐modified TGM PU with microtextures generated by casting on 800, 360, and 60 grit sandpaper, annotated by low, medium, and high roughness, respectively. (b) Laser scanning microscopy images of TGM PU (smooth) and TGM PU (textured) with increased surface roughness. (c) Quantification of the surface roughness between TGM PU (smooth) and TGM PU (textured). (d) Epi‐illumination images of SARS‐CoV‐2 spike protein‐FITC adsorbed on TGM PU (smooth) and TGM PU (textured) over a 2‐hour incubation period. (e) Quantification of the amount of bound SARS‐CoV‐2 spike protein in (d) based on FITC fluorescence intensity. (f and g) The amount of SARS‐CoV‐2 spike protein (f) and VLP (g) recovered in the eluent after being rinsed off from the TGM PU (smooth) and TGM PU (textured) as quantified by ELISA. (h) Reduced CoF in TGM PU (textured) as compared to TGM PU (smooth). *P < .05, **P < .01, ***P < .001. CoF, coefficient of friction; PU, polyurethane; VLP, virus‐like particles; WCA, water contact angle

While the TGM PU has been demonstrated to be suitable for use as the substrate material for FlexiPalms, we sought to ascertain its surface modifiability to enhance its functionality. To this end, we adopted a facile process to produce microtextures on the FlexiPalms surface by casting the TGM PU onto sandpaper. This increases the surface roughness which has been linked to augmented surface hydrophobicity and imparting of superhydrophobicity.^34^, ^35^ We cast the TGM PU on sandpaper with three levels of roughness—low (800 grit), medium (360 grit), and high (60 grit). Surface wettability measured using the sessile drop technique revealed that the WCA increased from 90.46 ± 4.00° to a maximum of 100.11 ± 0.46° on the medium roughness surface, corresponding to a 10.7% increase in hydrophobicity (Figure 8a). No significant change in the WCA was observed for the low roughness surface, while the high roughness surface displayed a reduction in the WCA owing to the reduced stability of the air plastron meniscus.^35^ As such, we proceeded to further evaluate the medium roughness surface, henceforth referred to as “TGM PU (textured),” while the raw TGM PU will subsequently be annotated as “TGM PU (smooth).” We characterized the surface topology of the samples by laser scanning microscopy which revealed an increase in the surface roughness from 9.45 ± 0.33 μm for TGM PU (smooth) to 13.75 ± 1.91 μm for TGM PU (textured) (Figure 8b,c).

This increased roughness is also postulated to reduce the contact between the virus and the material surface, hence reducing viral adsorption.^36^, ^37^ To confirm this, we conducted the protein adsorption study comparing the amount of recombinant SARS‐CoV‐2 spike protein binding on the TGM PU (smooth) and TGM PU (textured) surfaces. Indeed, the TGM PU (textured) (0.47 ± 0.07 A.U.) displayed a 53% reduction in the spike protein binding as compared to TGM PU (smooth) (1.00 ± 0.04 A.U.) (Figure 8d‐e). ELISA assay showed a similar reduction in spike protein binding in TGM PU (textured) (78 ± 12 ng) (Figure 8f). There was a corresponding 45% reduction in VLP binding in TGM PU (textured) (0.85 ± 0.13 x10^8^ particles) as compared to TGM PU (smooth) (1.55 ± 0.17 x10^8^ particles) (Figure 8g).

From general user feedback, some participants commented on the FlexiPalms being “sticky” with some restriction to sliding motion when two surfaces of the FlexiPalms are rubbed against each other. Increasing the surface roughness was expected to reduce the pairwise adhesiveness between the PU surfaces through reduction in contact area.^38^ From the inclined plane testing method, increasing the surface roughness reduced the static CoF by 55% from 2.09 for the TGM PU (smooth) to 0.93 for the TGM PU (textured) (Figure 8h). Through these results, we have demonstrated the surface modifiability of our FlexiPalms for improved functionality.

## 4 DISCUSSION

The use of PPE has been a stalwart addition to the fight against COVID‐19 in managing community transmission. To this end, tremendous effort has gone into sourcing for PPE,^39^ developing alternative PPEs,^40^, ^41^, ^42^, ^43^ addressing environmental sustainability,^44^ and understanding the role of PPE donning and doffing on cross‐contamination.^30^ We acknowledge that there is mixed opinion on the relevance of gloves usage for protection against the SARS‐CoV‐2 virus.^45^, ^46^, ^47^, ^48^ However, there is evidence that the SARS‐CoV‐2 virus can spread not only through aerosols but also through fomite transmission. Altogether, this warrants additional attention to hand‐targeted PPE to address the existing concerns that limit the wearing of gloves in the community. To this end, we designed and fabricated FlexiPalm, a novel hand‐targeted PPE, which demonstrated favorable mechanical properties, hydrophobicity, breathability, and minimal viral protein adsorption.

In the mechanical characterization, we compared commercially available PU materials to conventional glove materials (nitrile, latex, and LDPE). TGM PU exhibited superior ultimate tensile strength and elongation which accord enhanced mechanical durability and hand conformity. Despite having a considerably lower Young's modulus compared to LDPE, TGM PU showed a higher Young's modulus than latex, which suggests they might be slightly more rigid than medical gloves. PU elastomers comprise linear segmented copolymers with a relatively flexible polyol soft segment, and a relatively hard and stiff diisocyanate hard segment.^49^ PU derives its high elasticity from the rubber‐like matrix containing hard microdomains, while its high tensile strength can be attributed to hard microdomains physically cross‐linked through hydrogen bonding and dispersion forces, which act as a filler‐like reinforcement for the soft segment.^50^ High rigidity may impede hand motion and dexterity, but this is not the case evident from user feedback in our pilot study with FlexiPalms. With regard to the skin adhesion, we prioritized user comfort and pain infliction as criteria in assessing the tested materials. While all three PU materials (TGM PU, OPS PU and CXS PU) showed comparable levels of adhesiveness, the TGM PU was found to be the lowest, hence making it the least likely to induce dermal damage^51^ and inflict pain.^25^ In addition, the selected TGM PU material is a commercial transparent film material that is inherently designed to provide ample adhesiveness for prolonged wear without inducing skin damage during removal. Even though TGM PU was selected based on its lowest peel strength, the adhesiveness may be lowered if necessary, by replacing the acrylate adhesive with a silicone‐based adhesive, which has been shown to remove the least amount of stratum corneum alluding to reduced dermal damages.^25^ This may be applicable to users with underlying skin conditions. On the other hand, OPS PU and CXG PU with higher peel strengths may be substituted if added adhesiveness is required, especially for users with sweaty palms or high intensity usage.^52^

Fomite transmission of the SARS‐CoV‐2 virus occurs as a result of infectious respiratory aerosols being expelled and settled on surfaces. Hence, water repellent surfaces have the potential to reduce viral protein contamination by reducing their contact with the protein‐containing droplets.^53^ The TGM PU was found to be marginally more hydrophobic than nitrile and latex. To further improve its water repellency, the TGM PU may be modified to achieve superhydrophobicity (WCA > 150°) either by adjusting its chemical composition^54^, ^55^ or by introducing microtextures.^56^ Superhydrophobicity may also confer self‐cleaning properties to the FlexiPalms.^57^ TGM PU‐based FlexiPalms with high water vapor transmission will reduce the accumulation of moisture on the palms and increase user comfort, especially with prolonged use. In the event that there is still excessive sweat accumulation under the FlexiPalms due to severe palmar hyperhidrosis (sweaty palms), the porosity of the TGM PU material may be increased to make it more breathable,^58^ without compromising other factors including hydrophobicity and penetration to viruses. With regard to viral adsorption, FlexiPalms displayed a lower binding of SARS‐CoV‐2 spike protein and VLP compared to artificial human skin, which illustrates the ability of FlexiPalms to protect bare hands from viral contamination. PU contains carbonyl groups which are relatively weak Lewis‐base functional groups that are capable of resisting protein adsorption by hydrogen‐bonding to water strongly, preventing proteins from displacing the interphase water and entering the adsorbed state.^59^ While this displays the FlexiPalms' superior antifouling trait, there is still a risk of self‐inoculation of SARS‐CoV‐2 virus from FlexiPalms‐to‐face contacts. Further optimization of FlexiPalms substrate material in this direction shall be explored to supplement the mitigation of the fomite transmission such as the incorporation of antimicrobial additives.^60^

Although our pilot study involved only 41 participants, we have established a general receptiveness of the users to FlexiPalms and demonstrated the capability of FlexiPalms in handling and holding up to a variety of activities. We also collected astute feedback and additional design considerations for future refinement of the prototype. In the next phase of our study to assess the basic functionality of FlexiPalms, we will recruit a larger number of participants from a variety of demographics, which will also serve to reduce biases of the data. Furthermore, our current tasks in the pilot study are based on the GRASP rehabilitation exercises which only target activities of daily living that are more static in nature. We will expand the activities to include tasks involving more intense and long‐term usage with the hand such as those listed in the Katz index of independence in activities of daily living^61^ or negotiating complex tasks such as the Perdue pegboard test.^62^ To have a more comprehensive characterization of the ability of FlexiPalms to guard against the SARS‐CoV‐2 virus as well as its applicability in the clinical setting, we will also include medical personnel and patients from hospitals where the participants have an elevated risk of exposure to the SARS‐CoV‐2 virus. Lastly, we note that our exclusion criteria for the pilot study are people with open wounds and skin conditions (eczema), so people with such skin conditions are advised to use FlexiPalms with caution. Nonetheless, we verified our material to be a nonirritant from in vitro skin irritation tests.

Subsequently, we tested the surface modifiability of the TGM PU by creating microtextures on the TGM PU surfaces which remarkably yielded a significant improvement in the functionality of the FlexiPalms. We found an increase in wettability which may be attributed to the textured surface topology producing discrete three‐phase (solid‐air‐liquid) contact points that have been shown to display extremely low surface adhesion due to the trapping of an air plastron which reduces the liquid‐solid contact area.^63^ Concomitantly, the reduced contact between the virus and the microtextured surface resulted in a reduction in viral adsorption.^36^, ^37^ We also observed a reduction in adhesive friction post modification as increasing the surface roughness reduces the adhesion between elastic solids.^64^, ^65^

Our recommendation is to adopt the existing practice of regular handwashing and the use of sanitizer and disinfectant in tandem with the wearing of FlexiPalms. While usage of ethanol‐based disinfectant can swiftly inactivate the virion,^6^ a surge in the prevalence of hand dermatitis due to prolonged and frequent hand washing was observed.^66^ FlexiPalms can serve as a physical barrier against harsh disinfectants during the washing of hands, enabling users to maintain good hand hygiene practices with reduced risks of dermatitis, while reducing the occurrence of cross‐contamination. Furthermore, the suitability for use of FlexiPalms in the clinical setting, especially in surgical procedures involving the need for whole hand protection, remains to be evaluated. Nonetheless, general usage in wards and public areas by nurses, doctors, and auxiliary personnel can be considered for an added level of protection. FlexiPalms may also be adopted for use in other industries such as food handling, retail, and automotive, with facile modifications where necessary.

## 5 CONCLUSION

In summary, we developed FlexiPalms, a unique palmar‐side hand PPE for protection against fomite transmission of viruses. We showed that compared to conventional glove materials, the FlexiPalms demonstrated enhanced mechanical durability and breathability, comparable hydrophobicity, and displayed minimal adsorption of SARS‐CoV‐2 spike protein and VLP. Importantly, from the pilot study conducted, FlexiPalms presented good functionality and attained a high level of receptiveness from the participants as a new form of PPE for hand protection. The proof‐of‐concept surface modifiability of the FlexiPalm material suggests the possibility of further optimizing and improving the material towards superhydrophobicity and antimicrobicity. The FlexiPalms represent an effective guard against fomite transmission of viruses and complement existing pandemic control measures. An effective implementation of the FlexiPalms will bolster PPE sustainability and lead to a paradigm shift in the global management of COVID‐19 and other infectious diseases in general.

## AUTHOR CONTRIBUTIONS

**Justin Kok Soon Tan, Shang Wei Song, Jialiu Zeng, and Chih Hung Lo:** Conceptualization (equal); data curation (equal); formal analysis (equal); funding acquisition (equal); investigation (equal); methodology (equal); project administration (equal); resources (equal); software (equal); supervision (equal); validation (equal); visualization (equal); writing – original draft (equal); writing – review and editing (equal).

## CONFLICT OF INTERESTS

The authors declare no competing interest.

## Supporting information


**Figure S1.** Donning of FlexiPalms using the applicator prototype. (A) Components of the FlexiPalm applicator prototype. (B‐E) Donning procedure of FlexiPalms. (B) Peel the perforated backing material of FlexiPalms and align them with the palm‐shaped cut‐out before pasting onto the lid. (C) Close the applicator and expose the FlexiPalms adhesive for application. (D) Align hands through the palm‐shaped opening and exert pressure onto the FlexiPalms against the memory foam to break them along the perforated lines. (E) Successful donning of FlexiPalms onto users' hands upon withdrawing the hands from the applicator
**Figure S2.** A participant performing a list of activities of daily living with FlexiPalms during the pilot study. (A) List of activities of daily living based on the GRASP rehabilitation exercises. (B) Participants of the pilot study are instructed to perform the 11 activities of daily living (T1A to 7B) with FlexiPalms following the GRASP rehabilitation program for stroke patients
**Figure S3.** Post loading functionality assessment of FlexiPalm. (A‐C) Mechanical properties. Tensile strength (A), Young's modulus (B), and elongation at break (C) of FlexiPalm before and after loading. Non‐significance is indicated by ns

## Data Availability

All data are available from the corresponding author upon request.
