# Supplementary material for: A hand‐targeted auxiliary personal protective equipment for intervention of fomite transmission of viruses
Source: Bioeng Transl Med. 2022 Sep 29;8(5):e10411. doi: 10.1002/btm2.10411 (PMC9538315; doi:10.1002/btm2.10411)
Supplement: Supplementary file 1 — Figure S1. Donning of FlexiPalms using the applicator prototype. (A) Components of the FlexiPalm applicator prototype. (B‐E) Donning procedure of FlexiPalms. (B) Peel the perforated backing material of FlexiPalms and align them with the palm‐shaped cut‐out before pasting onto the lid. (C) Close the applicator and expose the FlexiPalms adhesive for application. (D) Align hands through the palm‐shaped opening and exert pressure onto the FlexiPalms against the memory foam to break them along the perforated lines. (E) Successful donning of FlexiPalms onto users' hands upon withdrawing the hands from the applicator Figure S2. A participant performing a list of activities of daily living with FlexiPalms during the pilot study. (A) List of activities of daily living based on the GRASP rehabilitation exercises. (B) Participants of the pilot study are instructed to perform the 11 activities of daily living (T1A to 7B) with FlexiPalms following the GRASP rehabilitation program for stroke patients Figure S3. Post loading functionality assessment of FlexiPalm. (A‐C) Mechanical properties. Tensile strength (A), Young's modulus (B), and elongation at break (C) of FlexiPalm before and after loading. Non‐significance is indicated by ns [file BTM2-8-e10411-s001.docx]

**Supplementary Information**


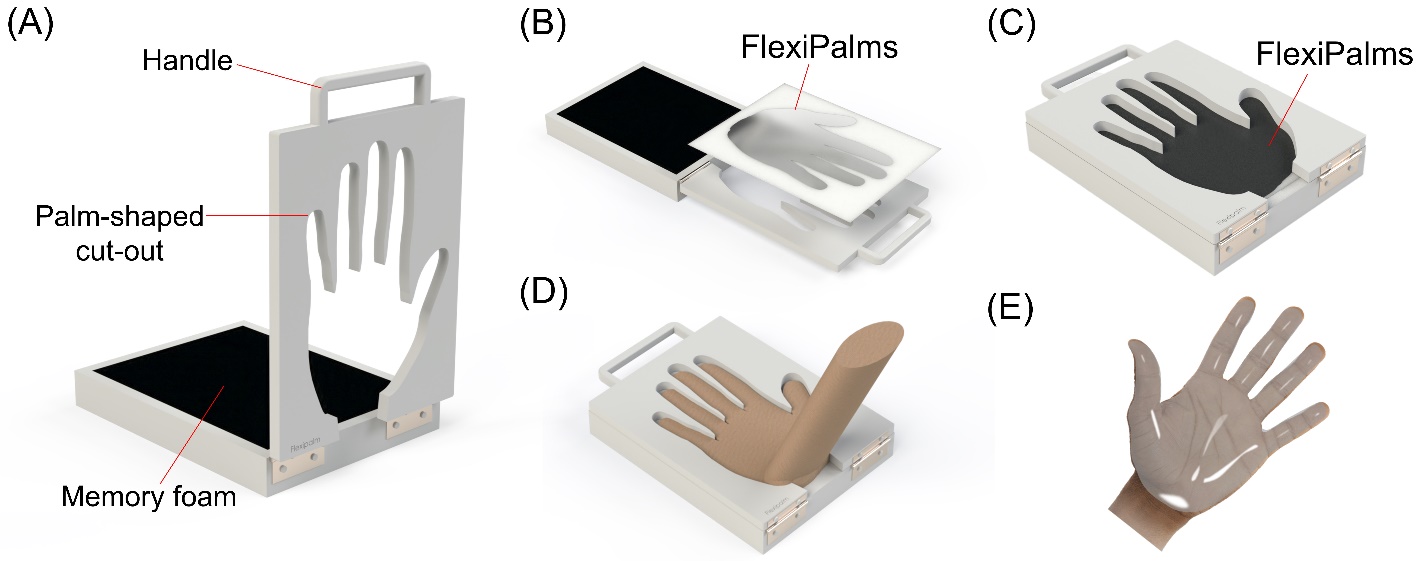


**Fig. S1. Donning of FlexiPalms using the applicator prototype.** (A) Components of the FlexiPalm applicator prototype. (B-E) Donning procedure of FlexiPalms. (B) Peel the perforated backing material of FlexiPalms and align them with the palm-shaped cut-out before pasting onto the lid. (C) Close the applicator and expose the FlexiPalms adhesive for application. (D) Align hands through the palm-shaped opening and exert pressure onto the FlexiPalms against the memory foam to break them along the perforated lines. (E) Successful donning of FlexiPalms onto users’ hands upon withdrawing the hands from the applicator.


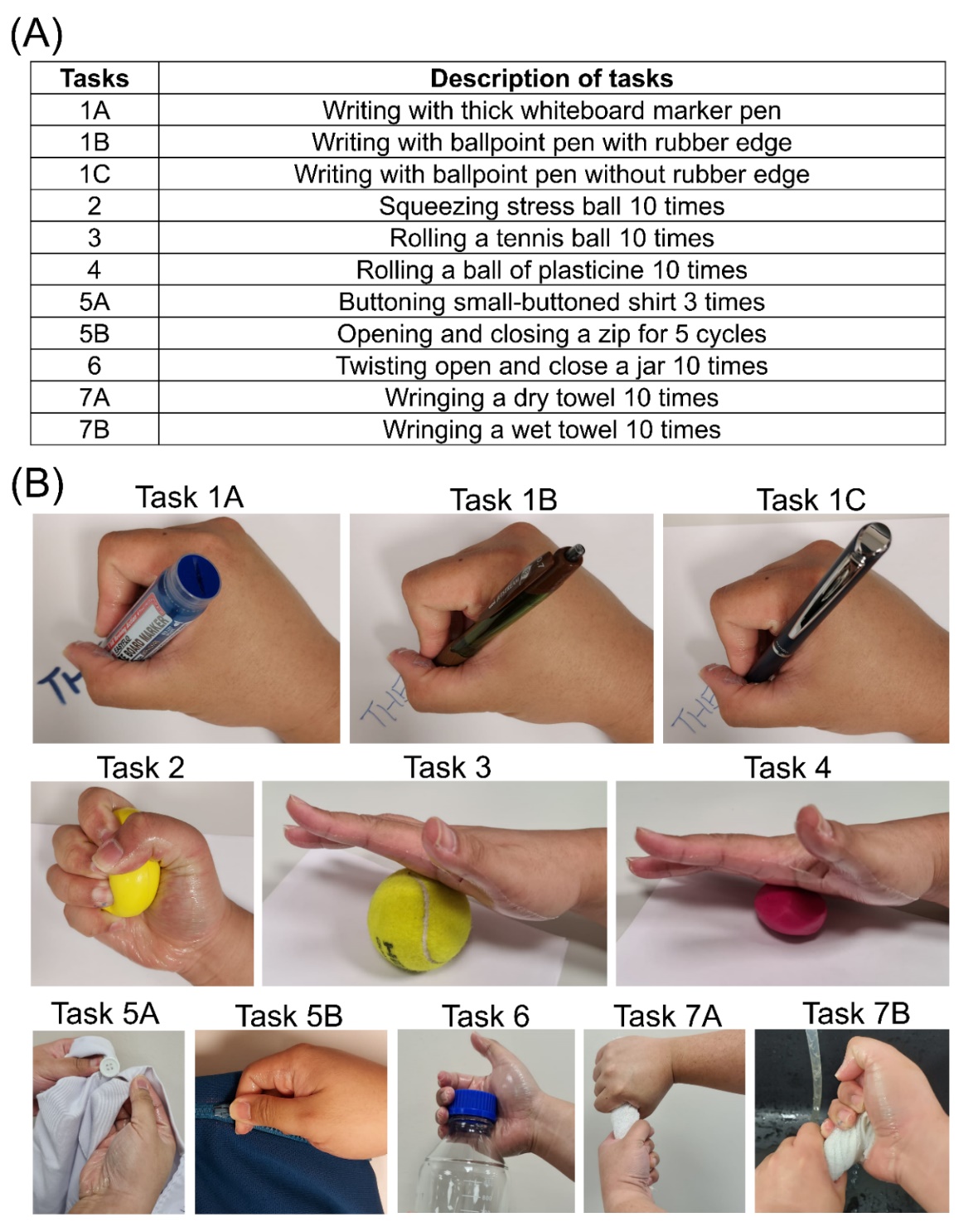


**Fig. S2. A participant performing a list of activities of daily living with FlexiPalms during the pilot study.** (A) List of activities of daily living based on the GRASP rehabilitation exercises. (B) Participants of the pilot study are instructed to perform the 11 activities of daily living (1A to 7B) with FlexiPalms following the GRASP rehabilitation program for stroke patients.


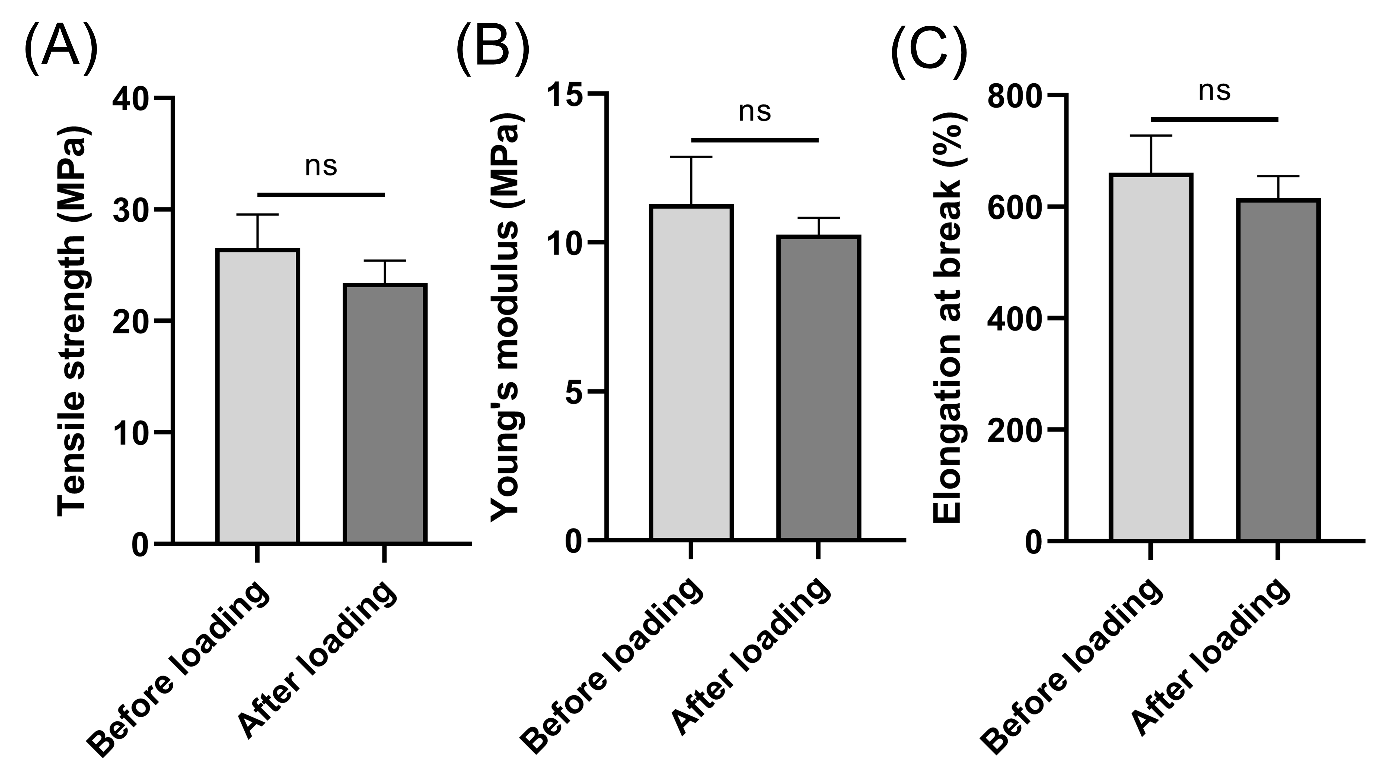


**Fig. S3. Post loading functionality assessment of FlexiPalm.** (A-C) Mechanical properties, tensile strength (A), Young’s modulus (B), and elongation at break (C) of FlexiPalm before and after loading. Non-significance is indicated by ns.
